# ‘I Have Never Been in That Kind of All‐Consuming Pain … I Did Not Know What Else to Do’: The Journey to Hospital Admission With Low Back Pain From the Perspectives of Patients

**DOI:** 10.1111/hex.70269

**Published:** 2025-04-23

**Authors:** Joseph F. Orlando, Matthew Beard, Anne L. J. Burke, Michelle Guerin, Saravana Kumar

**Affiliations:** ^1^ IIMPACT in Health, Allied Health and Human Performance University of South Australia Adelaide Australia; ^2^ Central Adelaide Local Health Network SA Health Adelaide Australia; ^3^ Commission on Excellence and Innovation in Health Government of South Australia Adelaide Australia; ^4^ School of Psychology, Faculty of Health and Medical Sciences University of Adelaide Adelaide Australia

**Keywords:** delivery of healthcare, determination of healthcare needs, hospitalisation, low back pain, qualitative research

## Abstract

**Background:**

There is a need to better understand patient factors contributing to low back pain (LBP)‐related hospitalisations to improve health service delivery and reduce avoidable admissions.

**Objective:**

This study explored the experiences of people with LBP leading up to and resulting in hospital admission.

**Design:**

Qualitative descriptive methodology using semi‐structured interviews.

**Setting and Participants:**

Patients admitted with non‐specific LBP at a large Australian tertiary public hospital meeting specific inclusion and exclusion criteria were pragmatically identified and recruited, and they consented to participate. Interviews were audio‐recorded, and thematic analysis generated codes and themes from the data.

**Results:**

Sixteen patients consented to participate. The cohort had an average age of 52 years (SD = 17) and had an average hospital length of stay of 13 days (SD = 10.8, range = 2–31). Two themes were identified. The first related to the impact of LBP on patients' lives, with sub‐themes including difficulty coping with pain, difficulty managing daily activities and escalating distress. The second theme related to unmet treatment needs, with sub‐themes including ineffectiveness of past treatments, inability to access timely and affordable community healthcare, and expectations for emergency care.

**Conclusion:**

Patients impacted by severe LBP regarded hospitals as a last resort for management of pain when community healthcare was perceived to be ineffective or inaccessible. This study highlights the practice gaps before hospitalisation for LBP from the perspectives of patients and the need to improve the delivery and access of healthcare for this condition.

**Patient or Public Contribution:**

This study sought insights from patients with low back pain (LBP) who were admitted to hospital with LBP. The findings will inform stakeholders, including consumers, on the co‐design of an optimal system of care to improve the delivery and access of health services for LBP. All patients were offered the opportunity to review a summary of the study's results.

## 1 Introduction

Hospitals worldwide face ongoing challenges to meet increasing healthcare demands due to ageing populations, rise in non‐communicable diseases, hospital system inefficiencies and poor funding of and access to preventive healthcare in the community [1, 2]. This burden impacts hospital bed availability and patient flow, which can contribute to treatment delays and suboptimal care.

Low back pain (LBP) accounts for 4.4% of global emergency department (ED) presentations [3]. Although many patients attending an ED with LBP perceive severe pain to be a sign of serious pathology requiring urgent care [4], the prevalence of serious spinal pathologies in EDs is, in fact, low (0.7%–7.4%), with most cases representing non‐serious LBP [5]. A majority can be managed in the community in accordance with clinical guidelines [6, 7, 8].

The percentage of people hospitalised after presenting to the ED with LBP varies around the world. Australia, whose public hospital system provides universal healthcare, has some of the highest rates of admission for LBP, ranging from 17% to 53%, compared to a median 9.6% (IQR 3.3–25.2) across nine countries [9]. This care is provided at significant cost, which in New South Wales (NSW) was estimated at AUD$13,137 per admission with an average length of stay of nine nights [10]. Whilst some admissions with a provisional diagnosis of non‐specific LBP may subsequently be found to have serious pathology, non‐specific LBP and sciatica still represent a majority of cases (62%) [11]. By seeking hospital care for their pain, these patients are exposed to high rates of investigations and analgesics, including opioids [12, 13], leading to care that may be inconsistent with clinical guidelines [14, 15].

Understanding risk factors for hospitalisation for LBP is important for health systems worldwide, given the challenges of delivering care for LBP. Empirical studies highlight factors such as older age (> 65 years), ambulance arrival to ED, undergoing medical investigations in ED, and area of residence as being associated with hospitalisation in this patient cohort [16, 17]. Qualitative studies also offer insights into patient experiences of hospitalisation for LBP. In a NSW public hospital, themes included severe pain and helplessness, perceived need for validation and investigation, and the desire to regain function [18]. Similarly, in a Danish university hospital, themes included difficulty managing severe pain, frustration with diagnostic uncertainty and recovery concerns [19]. These studies had minor methodological limitations, including single‐site recruitment, small sample sizes and limited interview depth of analysis.

This study aimed to build on existing research by exploring the experiences of people with non‐specific LBP leading up to and resulting in hospital admissions. It investigated the motivations behind their decision to seek emergency care, which ultimately resulted in hospitalisation. Understanding these experiences may help identify unmet clinical needs from patients' perspectives and inform improvements in LBP care delivery.

## 2 Materials and Methods

### 2.1 Study Design

Qualitative descriptive methodology was undertaken to summarise the perspectives of patients admitted for non‐specific LBP to highlight shared experiences of these events [20]. It is the least theoretical of all qualitative research methodologies and is an ideal framework to elicit a broad overview of the phenomenon of interest and obtain rich descriptions of patients' perspectives when little is known about the topic [21]. This study was informed by and reported using the COREQ checklist [22]. Ethics approval was granted by the Human Research Ethics Committees of the Central Adelaide Local Health Network (#15482) and the University of South Australia (#205112).

### 2.2 Sample

The target sample was patients admitted to the Royal Adelaide Hospital in Adelaide, South Australia, with a primary diagnosis of non‐specific LBP or sciatica. The public hospital has an 800‐bed capacity with an estimated 85,000 inpatient admissions per year. Patients were included in the study if they were adults ≥ 18 years of age; admitted to a general medicine or spinal inpatient ward for a minimum of 24 h; had an admission code for non‐specific LBP or sciatica (International Classification of Diseases: ICD‐10 M54.3, M54.4 and M54.5) and had conversational English. Acute, subacute and persistent pain types were included. Exclusion criteria included: being < 18 years of age; discharge delays secondary to medical conditions not related to LBP; having been diagnosed with serious spinal pathologies (i.e., fracture, cauda equine syndrome, infection and neoplasm), specific‐spinal diagnoses (i.e., radiculopathy with neurological loss) or back pain secondary to non‐spinal pathologies (i.e., visceral, vascular, urogenital and hip/pelvis); having other primary diagnoses and having undergone spinal surgery in the past 6 months. Patients were also excluded if they did not have conversational English or were unable to participate for other reasons (e.g., in police custody, detained under the provisions of a mental health act, and under the influence of illicit drugs or alcohol) to ensure all patients could provide informed consent to independently and voluntarily participate in the study.

### 2.3 Recruitment

Patients who met the inclusion criteria were pragmatically identified to participate in this research study by clinical staff working on the general medicine and spinal inpatient wards and involved in the care of patients with LBP. Clinical staff gained verbal consent from patients to pass on their details to the principal investigator (J.F.O.), who was not involved in patient care. The investigator approached patients face‐to‐face within their hospital rooms and formally recruited and obtained written consent to participate in the study. A well‐recognised form of sampling in qualitative research, purposive sampling, is ideal in selecting participants who are well‐informed of the phenomenon through knowledge or lived experience and have a ‘story to tell’ [23]. As part of purposive sampling, quota sampling of patients from different ages, social and cultural backgrounds, and duration of LBP formed part of the recruitment. For this study, the sample size (*n* = 15–18) was determined based on a recent systematic review examining sample size saturation in qualitative studies [24] and previous research on people with LBP in the hospital [4, 18]. Data collection was ceased when data saturation was reached by the research team. Patients had no prior knowledge or relationship with the investigator, but his clinical experiences and research interests in improving the delivery of care for LBP were openly disclosed.

### 2.4 Data Collection

Semi‐structured interviews were undertaken over 12 months between April 2023 and March 2024. A mutually convenient time was established to conduct the semi‐structured interviews face‐to‐face during the admission within the privacy of patients' hospital rooms. Interviews were conducted by the principal investigator using an interview guide with open questions and prompts that encouraged free‐flowing conversations (Table 1). The interviews explored patients' pain stories, experiences of healthcare before hospital presentation, personal and contextual factors related to pain, and the journeys to hospitalisation. The interviews were audio‐recorded, transcribed verbatim and de‐identified. Field notes were recorded in a journal. Additional patient data was obtained from the electronic medical records, including demographic information and clinical activity relating to the admission.

**Table 1:** Interview guide.

Can you tell me about your pain story?
Tell me about the care you received before coming to the hospital.
Did you feel in control of your symptoms?
Can you describe how pain impacted you, such as activity, work, life roles, relationships, mood, etc.?
What has been your experience of being in the hospital?
What was the reason you came to the hospital for your back pain, and what were your expectations?
What was the reason for you being admitted to the hospital?
Were you given other options to manage pain other than being admitted to the hospital?
If you could make suggestions on how the health system can improve the care for people with back pain, what would you recommend?

### 2.5 Data Analysis

Data from the semi‐structured interviews was managed using qualitative analysis software, NVivo version 14 (QSR International Pty Ltd., Melbourne, Australia). Thematic analysis was utilised using six phases: initial reading and familiarisation of the data, systematic generation of initial codes, searching for and organising codes into themes, reviewing themes for their appropriateness against the coded extracts, naming and analysing how themes are defined, and producing the report [25]. Themes identify key elements of data as they relate to the research topic. A thematic map of analysis was developed and reviewed to describe the dataset and account for the researchers' judgements in analysing the important messages from patients' perspectives on the events that preceded their hospitalisation for LBP [25].

### 2.6 Research Rigour

Rigour strategies were employed during the analysis process. Credibility was maintained through data triangulation using multiple investigators (J.F.O., S.K. and M.G.) to code transcripts and identify, develop and revise themes from the data. Patients were offered the opportunity to review a summary of the study's results and provide feedback on these findings. Dependability and confirmability were ensured by having an audit trail of decision‐making throughout the research process, which was then peer‐reviewed by an independent research group. Transferability was established by detailing the contextual information, behaviours and experiences of patients to extrapolate findings. Reflexivity was applied by the principal investigator, keeping a diary of his subjective responses through the research process [26, 27].

It is important to acknowledge and reflect on possible assumptions and experiences of the research team that may have influenced data collection and analysis. The principal investigator is a physiotherapist with clinical experience in managing LBP in outpatient, inpatient and ED settings, and this influenced his decision to undertake this study. The research team are advocates for evidence‐based practice of LBP as outlined by clinical care standards [6], which support community healthcare in the majority of cases. These beliefs were regularly discussed and documented in fortnightly meetings during data collection and analysis. The principal investigator had knowledge and training on qualitative principles before conducting the interviews through workshops held by the University of South Australia. Before interviews being conducted, the principal investigator underwent training with the supervisory team to practise interview techniques by conducting mock interviews and pilot testing the interview guide. Feedback was provided by the supervisory team. One member of the research team (S.K.) in particular is an experienced qualitative researcher who provided the principal investigator with expert mentorship during the data collection and analysis phases of the research.

## 3 Results

A total of 21 patients were invited, and 16 consented to participate. Three patients declined whilst in the company of family and in preparation for hospital discharge, and two declined, citing anxiety during the hospital admission. Table 2 provides an overview of the demographic characteristics and clinical features of patients. The majority of patients were male with an acute episode of LBP and averaged 13 days (SD 10.8, range 2–31) in hospital.

**Table 2:** Study patient characteristics.

	*n*	%
Age, mean ± SD (range) = 52.4 years ± 17.0 (22–74)		
Sex		
Male	10	63
Female	6	38
Residence		
Metropolitan	12	75
Regional	4	25
Marital status		
Single	4	25
Married/de facto	11	69
Widowed	1	6
Non‐English‐speaking background		
No	11	69
Yes	5	31
Highest level of education		
Secondary	13	81
Tertiary	3	19
Employment status		
Full‐time	6	38
Part‐time	2	13
Pension (age/disability)	6	38
Student	1	6
Healthcare funding		
Public	11	69
Private health insurance	4	25
Worker compensation	1	6
Diagnosis		
LBP	11	69
LBP with sciatica	5	31
Pain duration		
Acute < 6 weeks	8	50
Subacute 6–12 weeks	2	13
Persistent > 12 weeks	6	38
Medical history		
Previous LBP episode	11	69
Anxiety/depression	10	63
CCI ≥ 2	4	25
Healthcare utilisation reported before hospital presentation		
General practitioner visit	11	69
Opioid analgesic prescription	8	50
Physiotherapist/allied health visit	7	44
Spinal imaging	5	31
Specialist consultation	0	0
Previous ED presentation for LBP within the last 7 days	5	31
Arrival mode to the ED		
Ambulance	10	63
Private car	6	38
Admitting inpatient ward		
General medicine	14	88
Spinal unit	2	13
Discharge destination		
Home	10	63
Country hospital	2	13
Rehabilitation hospital	2	13
Respite facility	2	13
Days in hospital, mean ± SD (range) = 13 days ± 10.8 (2–31)		

Abbreviations: CCI, Charlson Comorbidity Index; ED, emergency department; LBP, low back pain; SD, standard deviation.

Interviews were conducted in person (*n* = 16) by the principal investigator during the hospital admission within each patient's private room. Only the investigator and patients were present during the interviews. Each interview lasted 20–30 min. There were two overarching themes: the high impact of LBP and unmet treatment needs. Six sub‐themes were identified, three under each theme, respectively: difficulty coping with pain, difficulty managing daily activities, escalating distress, ineffectiveness of previous treatments, inability to access timely and affordable community healthcare, and expectations for emergency care.

### 3.1 Theme 1: The High Impact of LBP

Patients reported experiencing a high negative impact from LBP across a range of aspects of daily living in the lead up to their hospital admissions. They described the severity of symptoms, the impact on physical function and emotional well‐being, and their struggle to manage the impacts of pain.

#### 3.1.1 Sub‐Theme 1.1: Difficulty Coping With Pain

Patients described experiencing extremely severe levels of pain. This was reported by all patients irrespective of their demographic characteristics, pain location (low back and/or leg) or symptom duration. They also described feeling overwhelmed by their inability to experience relief from symptoms or to control them.

‘The pain continued to get out of control. It was more frequent, and not manageable. I know it sounds so overdramatic, but I have never been in that kind of all‐consuming pain that was inescapable…. It was the most intense thing that I'd felt. So, I honestly don't know how I would have kept going.’

[P9]

‘It was very extreme pain. I would be writhing, clutching my leg, crying, screaming for the entire two hours. And then in two more hours it would happen again.’

[P1]

Many patients characterised their LBP as the worst pain they had ever experienced. They compared their current episode to past pain experiences, highlighting their difficulty managing pain within the present.

‘I was crying with pain. It was the worst thing. I jokingly said, “I'd rather go through my heart attack.” The way I had my heart attack … to reenact that again. That wasn't half as bad.’

[P13]

‘I have had multiple unmedicated births and I would do that any day repeatedly over this back pain.’

[P9]

#### 3.1.2 Sub‐Theme 1.2: Difficulty Managing Daily Activities

Patients said that they had struggled to manage daily activities at home in the immediate day(s) before hospital admission. They reported spending long periods resting in bed due to difficulty moving with pain. Inability to attend to personal care and move about their homes was described as a ‘*breaking point’* [P9] for what could be tolerated to function at home.

‘I couldn't keep going at the house by myself because we have stairs. I couldn't get out of bed because I couldn't move. My partner had to look after me. I can't look after myself; can't do the simple things like wiping my own bottom or put socks on…. The pain was at a point where I couldn't manage it at home. Unfortunately, that's just not a reality that you can live. You can't just stay in your bed.’

[P7]

Patients reported that they called on family members, where possible, to assist with essential household and/or caregiving tasks that they felt unable to perform themselves. However, they also said that this support was often difficult for them to accept due to the perceived burden it placed on the family.

‘I can't take care of my children. I can't work, I can't drive, I can't walk, I can't clean, I can't cook. I had to get my mum to take my kids because I'm a single parent.’

[P12]

‘We have three children at home and my partner has had to stay with them so that I could come to hospital.… I had to do it, which was tough.’

[P9]

It was especially challenging for older people and those living alone without family support. Older people were managing daily functions from a low baseline whilst also managing their comorbidities. Patients stated that their independence and safety at home were suddenly compromised by the addition of LBP.

‘I've got diabetes, high blood pressure, and of course this leg pain…. Over the week it progressively got worse and worse. All I did at home was lay in bed all day. I couldn't really function at all. Even making a cup of coffee was pretty much beyond me. I had probably had six Chupa Chups, a Chomp bar and Curly Wurlys, about three of them. That's all I ate for about four days.’

[P10]

‘I was unable to shower or, I suppose, self‐care for myself. Even going to the toilet was hard.’

[P13]

#### 3.1.3 Sub‐Theme 1.3: Escalating Distress

High levels of distress relating to LBP were reported by patients. Uncertainty around clinical diagnosis and prognosis (e.g., ‘*Is it something bad? You always think the worse’* [P14]) was reported as a key driver for seeking urgent care. Patients described feeling fearful that symptoms were indicative of serious pathology (e.g., ‘*I thought it was gonna be cancer’* [P16]), that may have potentially fatal outcomes (e.g., ‘*I thought I was going to die’* [P6]) or that they may require invasive treatments (e.g., ‘*I was very stressed that I'd have to go into surgery’* [P7]). Many attributed their distress to their perceived inability to control pain, which further increased their uncertainty surrounding their condition.

‘It got to the point where I just started saying, “I can't do this.” I'm usually a power‐through kind of person, but I could not handle that pain…. This was just so overwhelming. I was scared, I guess as well, because I didn't know what was happening.’

[P9]

Most patients also described concurrently dealing with other life stresses that were impacting their pain experiences (e.g., ‘*My mum passed away. So, I've had to deal with all the pain, the stress of my mum and everything’* [P5]). Younger patients discussed feeling stressed about their employment (e.g., ‘*Might have to try a different career. It really is life‐changing, back injuries’* [P11] and ‘*…the financial burden … taking your sick leave’* [P7]) and a self‐employed farmer was dealing with the stress of work whilst managing pain (e.g., ‘*I've got 15,000 sheep and 3,000 hectares of crop that I have to manage. I've gotta get up and keep on going’* [P8]). One student reported feeling overwhelmed by assessment deadlines, stating that ‘*My thesis is due in two months. I still have experiments to do. I've already had it extended’* [P1]. Older people described the burden of managing LBP alongside their comorbidities (e.g., ‘*I have many problems: my kidney and my heart, and I have so many, many problems’* [P2] and ‘*My diabetes is pretty much around 14, 15…. I don't seem to manage anything very well’* [P10]).

The long‐term impacts on mental health experienced by patients with a history of LBP (*n* = 11, 69%) or anxiety/depression (*n* = 10, 63%) contributed to the current pain and distress experienced. One patient, only during the interview, ‘*realised how much PTSD from the last (spinal) surgery’* [P7] was present and affecting her current episode of LBP. Another stated that with each pain flare he experienced, he ‘*…just feels like letting go (of life). I'm in that much pain’* [P6]. Another patient described feeling concerned about his capacity for recovery following the current episode of LBP.

‘It's definitely a mental setback—the ability to come back, get back into normal life. If I can do that pretty quickly, it's not too bad, but if I get housebound, that's when I start getting a little bit … not as happy.’

[P3]

### 3.2 Theme 2: Unmet Treatment Needs

Patients described experiencing unmet treatment needs for LBP before hospital admission. They asserted that previous treatments had been ineffective and that other care types were inaccessible and/or too costly for them to utilise. As such, they believed that their care needs could no longer be addressed in community healthcare. Some considered their treatment needs to be more urgent requiring emergency care.

#### 3.2.1 Sub‐Theme 2.1: Ineffectiveness of Previous Treatments

Many patients reported that they had sought care from their general practitioner (GP), physiotherapist and/or chiropractor, ranging from 1 week to 3 months before their hospital admission. Treatments included self‐management (physical activity, heat and meditation), physical therapies (massage, manipulative therapy, acupuncture and exercise), pharmacotherapies (paracetamol, non‐steroidal anti‐inflammatories, opioids, anticonvulsants and muscle relaxants), and spinal injection therapies. The effectiveness of specific treatments varied significantly between patients, but the common themes were overall perceived treatment ineffectiveness and lack of treatment options in the community, necessitating hospital presentation.

‘My physio and my GP both said, “If it doesn't get any better, your last resort is going to emergency.” I've done all of that. I had an epidural, I did two CT scans, I've been going to my physio, I've been doing everything.’

[P12]

‘I tried, I think in the span of a few days, I spoke to five GPs. I had two ambulance officers come out. I did not know what else to do. I didn't have a choice.’

[P9]

Patients' low pain self‐efficacy was linked to perceived treatment ineffectiveness. They described feeling as though they had exhausted their treatment options without experiencing sufficient improvements. One patient described escalating analgesic use, stating ‘*I needed to take them every six hours, to five hours, to four, then every three … with only mild relief’* [P1]. Another reported repeated treatment failures.

‘I went to the doctor and they gave me some pain killers, but it didn't do it any good. Then they gave me a needle into the buttock, but that didn't help me either. I was having hot baths, and ice packs. The pain was still there. I guess I was desperate…. I really needed pain relief.’

[P13]

Consequently, many patients felt dissatisfied with healthcare before hospital presentation. One patient [P4] described the health system as ‘*a bit broken’* as his treatment needs were unable to be addressed within allocated appointment times and he perceived the health professionals he consulted as lacking in ‘*compassion’*.

‘I went seeing two different doctors and they just almost brushed it off and they seem like you've got eight minutes there, they want you in and out. They didn't give you advice … and the fact that it would've been nice to know. I know everyone's different, but they could have just given me a brief idea of what I can and can't do.’

[P4]

#### 3.2.2 Sub‐Theme 2.2: Inability to Access Timely and Affordable Community Healthcare

Many patients reported that they were unable to get timely appointments with their regular GPs before presenting to the hospital. Patients were frustrated and disappointed, stating ‘*When I needed to go, my doctor wasn't available’* [P1], making it difficult to receive prompt care to manage their LBP. Some patients secured appointments with other available GPs, locum home visits or telehealth services, especially those that ‘*couldn't actually physically get in to see a doctor’* [P9] due to pain severity. The challenge of getting timely appointments was particularly difficult after‐hours, on weekends and in regional areas, leading to hospital presentations.

‘A few days after I'd been to the chiropractor, I tried to make an appointment at the doctors, but there were no vacancies for ten days. There's a shortage of doctors, and they're heavily booked. I thought, “Nah, can't handle this no more!”’

[P16]

The cost of GP appointments was also a barrier to accessing community healthcare. Many patients commented that they found it difficult to find a GP who did not charge out‐of‐pocket expenses. One patient said that he did not attend community healthcare at all before presenting to the hospital ED, stating ‘*Finances there were draining me, so I couldn't see a doctor if I wanted to’* [P10]. Imaging, medication prescriptions and allied health services all contributed to additional pain‐related costs, impacts that were experienced most profoundly by people without private health insurance and those from lower socioeconomic areas.

‘The thing that did hurt was it's no longer fully bulk‐billed, so every time I had to see them, every time it was getting worse or I needed to renew the prescriptions, I had to pay a gap. It hit the most when I had an MRI. Everywhere I called, they quoted me about $300. I'm a student. That's very difficult.’

[P1]

‘Cost is a lot, especially with the cost of living at the moment. Yeah, you're paying nearly $80 a visit for a physio. Sometimes, that can be twice a week. Then if you need to do extra things like hydrotherapy or I suppose Pilates to strengthen, it can be even more. Then again, medication on top of that. Illness can become quite costly.’

[P7]

#### 3.2.3 Sub‐Theme 2.3: Expectations for Emergency Care

At the point of attending a hospital ED, patients considered their treatment needs to be urgent and requiring emergency care. They felt their treatment could not be addressed in the community and that presentation to the hospital ED was required. Effective pain management was the most common treatment sort, specifically access to strong analgesics.

‘I knew that as soon as I got to hospital, I needed medicine. I couldn't wait to get there, and I couldn't wait to have those tables to get rid of the pain. I wouldn't have achieved much with the GP. I don't think he would have given me Endone. He may have said to get some physio. I made my own decision on what I needed, and did that.’

[P13]

Patients also expressed a desire for their pain management to be monitored in the hospital setting.

‘I feel like I'm in a safer space for that pain management program to be monitored. I also just think getting that physio, be able to see what I can do in here, and try and be able to move because yeah, I just don't think I could do it at home.’

[P7]

Urgent access to specialist care was the other key expectation that patients sought when attending the hospital. Some patients reported being on waitlists for specialist outpatient appointments and said that they hoped to expedite their care by attending the ED because ‘*there's no other way for me to get into see a spinal doctor’* [P12]. According to some patients, the ED treating clinicians decided to investigate and admit patients under specialist teams (e.g., ‘*I was basically rushed down here for an emergency MRI’* [P8] and ‘*They came up with a management plan and they decided to admit me’* [P1]). Other patients felt they had to advocate for investigations to be conducted, leading to conflict with clinical staff.

‘You guys need to look at it properly and figure out what's going on and get me admitted. Write a letter to the Spinal Unit and say, “This woman's coming in agony, she can't do anything. Can we get her looked at sooner!”’

[P12]

## 4 Discussion

This study was undertaken to better understand patients' perspectives about their pain journeys before and leading to hospitalisation for LBP. Patients reported that pain had a significant impact on their lives, affecting their physical function and emotional well‐being. At the point of presenting to a hospital ED, they no longer perceived they had the capacity to manage their pain, asserting that their treatment expectations had been unmet by community healthcare services, which they considered to be ineffective and inaccessible. As a result, they sought urgent care from a hospital ED and were subsequently admitted.

This study's findings align with the broader qualitative literature on the lived experience of LBP. A meta‐ethnography of 38 qualitative studies described the experiences of people with severe chronic LBP as being marked by wide‐ranging distress and loss, which affected them on multiple levels, and poor experiences and interactions with the healthcare system [28]. Importantly, most of this literature focused on community settings. In contrast, our study offers several novel insights. First, as it was conducted during patients' hospital admissions for LBP, it captured their experiences in real time, providing a unique perspective on their decision‐making and expectations of emergency care. Second, these experiences were recorded at the point where patients transitioned from the community into the hospital setting. Third, by focusing on those who sought emergency care and required hospital admission, our study sheds light on the perceived gaps in outpatient or community healthcare that may contribute to hospital presentations, highlighting potential areas for improvement in LBP management.

The high impact of LBP on patients' lives contributed to patients' decision‐making to present to the hospital. Previous qualitative studies on patients with LBP in hospital settings described this impact as high perceived pain and disability [4, 18, 19, 29], external locus of control [18] and concern for one's condition [19, 29, 30]. A cohort with unspecified chronic pain conditions presenting to an ED was characterised by escalating distress when patient needs outweighed capacity to manage pain [31]. Low quality of life and reduced social supports further impacted pain coping mechanisms in people admitted to the hospital, especially with chronic LBP [19]. This is consistent with our study's finding that patients admitted to the hospital with LBP were burdened by the high impact of pain that surpassed their threshold to manage using their available resources. Low health literacy, whilst not explored in this study, has also been linked to higher utilisation of emergency care services and lower use of preventive care in patients with LBP [32]. This demonstrates how patient and contextual factors shape the lived experience of pain and influence patient coping and healthcare‐seeking behaviours.

In this study, patients considered community healthcare services ineffective and inaccessible. Previous studies have found that patients with LBP present to EDs in anticipation of care that is more rapid, accessible and comprehensive compared to community healthcare [4, 30]. There are health system challenges that may be contributing to ED presentations in this cohort of patients. In Australia, as with other parts of the world, patients are increasingly left with out‐of‐pocket expenses when accessing community healthcare, an expense that is most difficult for low‐income earners to accommodate [33, 34, 35]. GP appointment times are short and insufficient for complex cases with severe or chronic LBP [36, 37]. Workforce shortages affect service and provider availability, especially in regional areas [38], making it difficult for patients with LBP to receive timely care. Whilst there have been calls to action to improve the delivery of high‐valued care for LBP and address the inequities of healthcare access [37, 38, 39], this practice gap remains unaddressed as evidenced by the experiences of patients in this study hospitalised with non‐specific LBP.

Patients in this study also felt their treatment needs and expectations were not addressed by community healthcare before hospitalisation. Managing patient factors within the broader biopsychosocial framework is often challenging for clinicians [40, 41] due to time and resource limitations, lack of training in psychosocial and non‐pharmacological management, and poor awareness of clinical guidelines [36, 42]. Consequently, management of LBP is overly reliant on diagnostic imaging and medication prescriptions and less on patient‐focused interventions delivered through education, reassurance, self‐management advice and support [15, 36]. Failure to deliver personalised care and reduce the impacts of pain can contribute to patient uncertainty [43], leading to poorer outcomes such as disability [44] and increased healthcare utilisation [45]. This study highlights the importance of patient‐centred care in the management of LBP through the perspectives of patients hospitalised with LBP. Further investigation is needed to determine if community healthcare can therefore help prevent LBP‐related hospital admissions in accordance with clinical guidelines [6, 7, 8].

This study did not explore the quality of care received by patients in the community before presentation to the hospital. Previous studies have investigated the perspectives of clinicians managing LBP in the community and found that even when management for LBP was delivered in accordance with clinical guidelines, potential discrepancies between the care provided by clinicians and patient expectations led to patient dissatisfaction and poor engagement with treatment [46, 47]. These studies highlight the importance of exploring patient expectations to enable shared decision‐making between clinicians and patients on an optimal treatment plan. This is relevant to this study's finding that patients admitted to the hospital with non‐specific LBP present with unmet treatment needs and expectations.

As with all research, this qualitative study has its strengths and limitations. This study recruited a diverse group of patients with different ages and socioeconomic and cultural backgrounds, living in different geographical regions, and with acute and chronic LBP. People with LBP whose hospital discharge was delayed secondary to medical conditions not related to LBP and people without conversational English were excluded, and their experiences and factors preceding hospitalisation were not captured. Other patients with LBP excluded from this study were those discharged from the ED and not admitted to the hospital, representing the largest cohort of patients with LBP in the ED setting. The study was conducted at a single location in a large South Australian pubic hospital. Interviews at different sites (regional or private hospitals) may have revealed additional findings. Interviews were conducted during hospital admissions, allowing for in‐depth descriptions of patient experiences that preceded hospitalisation; however, some patients were precluded from participation due to discomfort or availability whilst in the company of family or in preparation for hospital discharge. Interviewing patients following discharge would provide further reflections on their experiences.

## 5 Conclusion

Patient factors that preceded hospitalisation for non‐specific LBP included significant pain impact and unmet treatment needs and expectations. Patients with LBP reportedly regarded hospitals as a last resort for management of pain after community healthcare was perceived to be ineffective or inaccessible. This highlights an opportunity for the co‐design of community healthcare services with stakeholders, including consumers, to improve the delivery and access of care for LBP. Further investigation is required to determine whether certain LBP‐related hospital admissions can be prevented.

## Author Contributions

**Joseph F Orlando:** conceptualisation, methodology, writing – review and editing, writing – original draft, investigation, data curation, validation, formal analysis, project administration, funding acquisition. **Matthew Beard:** supervision, writing – review and editing, formal analysis, validation, funding acquisition. **Anne L J Burke:** formal analysis, writing – review and editing, validation. **Michelle Guerin:** conceptualisation, methodology, validation, formal analysis, supervision, writing – review and editing. **Saravana Kumar:** conceptualisation, methodology, validation, formal analysis, supervision, project administration, writing – review and editing, resources, funding acquisition, software.

## Ethics Statement

Ethics approval was granted by the Human Research Ethics Committees of the Central Adelaide Local Health Network (#15482) and the University of South Australia (#205112).

## Consent

Patients provided written consent to participate in this study.

## Conflicts of Interest

The authors declare no conflicts of interest.

## Data Availability

The data that support the findings of this study are available from the corresponding author upon reasonable request.
